# Psychometric Properties of the MDADI—A Preliminary Study of Whether Less is Truly More?

**DOI:** 10.1007/s00455-021-10281-9

**Published:** 2021-03-11

**Authors:** Daniel J. Lin, Jenan Altamimi, Kim Pearce, Janet A. Wilson, Joanne M. Patterson

**Affiliations:** 1 Translational and Clinical Research Institute, Newcastle University, 3rd Floor, William Leech Building, Medical School, Framlington Place, Newcastle Upon Tyne, NE2 4HH UK; 2 Freeman Hospital, Newcastle Upon Tyne Hospitals NHS Foundation Trust, Newcastle Upon Tyne, UK; 3 Faculty of Medical Sciences Graduate School, Newcastle University, Newcastle upon Tyne, UK; 4 Institute of Health & Society, Newcastle University, Newcastle Upon Tyne, UK; 5 School of Health Sciences, University of Liverpool, Liverpool, UK

**Keywords:** Dysphagia, Head and neck cancer, Quality of life, Patient-reported outcome, Factor analysis, Deglutition, Deglutition disorders

## Abstract

The MD Anderson Dysphagia Inventory (MDADI) is a 20-item dysphagia-specific QOL questionnaire with four subscales: global, emotional, functional, and physical. It is widely used in clinical practice and in research; however, its psychometric properties have been under-researched. We aim to evaluate the organisation of the MDADI subscales and identify any redundant items. The MDADI is a routinely collected outcome measure at two centres in northeast England. Questionnaires completed at three months following treatment were extracted from these existing databases. Factor analysis was carried out with the aim of reducing redundancy among the set of questionnaire items. Cases with missing values were excluded. A total of 196 complete patient questionnaires were used in factor analysis. A one-factor model accounted for around 50% of the total variance in item responses. The top five endorsed items (abbreviated by the questionnaire item keywords: Excluded, Irritate, Esteem, Social, and Why) in this one factor appeared in three (emotional, functional, and physical) of the four supposed MDADI subscales, i.e. global, emotional, functional, and physical. Our results suggest an overlap of three MDADI subscales across the top five endorsed items. The content of the top five questions all appear related to the psychosocial aspects of swallowing. This implies some redundancy of the items in the original subscales of the questionnaire. Using the most endorsed items, it appears feasible to abbreviate the 20-item MDADI questionnaire to a 5-item “MiniDADI” questionnaire, which is likely to have greater utility in routine clinical practice outside of research settings.

## Introduction

Dysphagia is a very common condition and treatment-related symptom in head and neck cancer (HNC) and is adversely affected by both surgical and non-surgical treatments [1, 2]. It is strongly associated with poorer quality of life (QOL) outcomes, with fundamental changes to eating pattern, social life, and family relationships [2–4]. Dysphagia, thus, remains a significant and serious concern in the long term [5, 6]. Research has consistently shown that swallowing is a top priority concern for HNC survivors [3].

Patient-reported outcomes (PRO) measure patients’ health-related QOL (HRQOL) at a single or multiple time points. They are used to collect information about patients’ experiences of symptoms, condition and QOL, enabling individual and group level monitoring of outcomes and identification of those in need of intervention. PROs need to have proven reliability and validity to ensure that they are fit for clinical purpose. A practical and effective PRO should measure the underlying constructs of the questionnaire with minimal response fatigue [7].

The MD Anderson dysphagia inventory (MDADI) is a self-administered dysphagia-specific QOL questionnaire [8], designed to capture patient perceived problems for those treated for HNC. The MDADI is the first valid and reliable tool that is concerned with dysphagia-specific QOL for HNC patients, making it one of the most widely used QOL questionnaires clinically and in research settings [9, 10]. The MDADI was used as a primary or a secondary endpoint in clinical trials, e.g. PATHOS, DARS and ORATOR [11–13], also in validating new scales [14] and in determining the feasibility and utility of interventions (for example, investigating the effectiveness of pre-treatment swallowing exercises on post-treatment swallowing QOL [15]), efficacy of acupuncture on swallowing-related QOL [16], the feasibility of cognitive-behavioural swallowing therapy [17], and the effectiveness of electrical stimulation on swallowing [18]. It was also used to report on swallowing function in longitudinal studies [5, 19, 20].

Having a tool that is both clinically relevant to patients and has good psychometric properties is essential. Initial MDADI concepts were, however, developed through focus groups of head and neck surgeons and speech pathologists from a single centre, few with personal experience of dysphagia. The phrasing of the questionnaire was subsequently refined in focus groups of HNC patients with dysphagia. The final questionnaire consists of 20 items (listed in Appendix A) and includes four subscales: global (one item), emotional (six items), functional (five items), and physical (eight items) [8]. The global assessment is scored individually, while the other items in each subscale are summed and the mean score is multiplied by 20 to obtain a score that ranges from 20 (extremely low functioning) to 100 (high functioning). The MDADI has good internal consistency reliability (Cronbach alpha coefficient = 0.96) and a test–retest reliability correlation ranging from 0.69 to 0.88 for all of its subscales. The MDADI also proved to be valid in terms of criterion and construct validity when compared against the Performance Status Scale (PSS) dysphagia measure and the Short Form Health Survey (SF-36) HRQOL tool, respectively [8].

To date, the psychometric properties of the MDADI including its construct validity have been under-researched. In the initial development of the scale, although the authors investigated the questionnaire’s reliability, content, criterion, and construct validity, further analysis to identify any item redundancy was not undertaken. The current MDADI questionnaire in its full length covers two full pages; it can be difficult to score for the non-expert and can be potentially cumbersome for patients to complete, thereby potentially reducing the accuracy of answers [21] especially when administered repeatedly on follow-ups and, particularly, when combined with other clinical assessments [7]. Patients are also now frequently asked to complete other PROs such as HRQOL questionnaires, where there might be a degree of overlap in the clinical scales being measured. Furthermore, some centres may be collecting these data remotely via telehealth, which can be time consuming for both the assessor and patient if applying the MDADI. Therefore, we aim to evaluate the underlying psychometric construct of the MDADI and identify any redundant items.

## Patients and Methods

### Databases

Three databases collated at two university teaching hospitals in northeast England were used for this analysis. These databases were set up for audit or research purposes and included the MDADI as part of a battery of swallowing outcome measures. Patients were consecutively and prospectively approached, and their data were anonymised when enrolled into the databases. The focus of the databases was 1) a service evaluation of functional outcomes for minimally invasive surgery (transoral laser microscopy (TLM) or transoral robotic surgery (TORS)), 2) a research database recording swallowing outcomes for non-surgical primary treatment (chemotherapy (CRT) or radiotherapy (RT)), and 3) a feeding tube audit comparing outcomes for those receiving a reactive nasogastric tube (NGT) or prophylactic radiologically inserted gastrostomy (RIG) tube for primary or adjuvant (chemo)radiotherapy.

### Questionnaire

The MDADI was routinely collected pre-treatment, three, and 12 months post-HNC treatment. Questionnaires completed at three months were extracted from these existing databases. This time point was chosen as it represented the greatest deterioration in post-treatment MDADI scores and contained a full range of the scale of responses. It therefore maximised coverage of most questionnaire items [3, 20]. In order to conduct factor analysis on the patient responses, a wide range of scores is desirable [22, 23]. We opted not to use data from the same patient twice (e.g. both three and 12-month time points), as their questionnaire interpretation and responses would be very similar. Each of the 20 MDADI items are rated on a 5-point Likert scale ranging from 1: “strongly agree” to 5: “strongly disagree”, except for questions abbreviated by “Conscious” and “Eat Out” (Appendix A) whose ratings are scored in reverse order ranging from 5: “strongly agree” to 1: “strongly disagree”. A composite MDADI score was generated by calculating the mean response for the 19 items (excluding the global question) making up the emotional, functional and physical subscales and multiplying the result by 20, resulting in a score ranging from 20 representing a low QOL function to 100 indicating high QOL function[8]. All MDADI question responses were inputted into the data extraction sheet for analysis.

### Factor Analysis

The data were analysed using IBM SPSS Statistics for Windows, version 24 (IBM Corp., Armonk, N.Y., USA). Multivariate factor analysis was carried out to examine relationships between multiple ordinal questionnaire items each measured on a Likert scale and collected from the 3-month MDADI questionnaire. By exploring the structure of the data in this manner, we were informed as to whether item reduction was viable.

It is recommended that at least ten completed questionnaires per questionnaire item are used for factor analysis implementation [22, 24, 25].

For this study, factor analysis was carried out using the most recommended and utilised combinations of options [24], i.e. (i) principal axis factoring (PAF) and (ii) principal components extraction. Principal axis factoring is, strictly, a “factor analysis” method whereas factor analysis with principal components extraction is often termed “PCA”. Although different in their mathematical derivation, factor analysis and PCA share a common aim: to identify the underlying dimensions in the data. It is, however, acknowledged that there is no guarantee that factor analysis and PCA will result in the same solution [23, 26]. Formally, the dimensions are called “factors” (in factor analysis) and “components” (in PCA); however, for readability, we have termed both entities “factors”.

In a factor analysis, the total number of factors equals the number of items in the questionnaire. Each factor captures a proportion of the overall variance in the observed items. Factors are output in the order of how much variation they explain with the eigenvalue representing the variance explained by a particular factor. The first factor is, thus, the most important and accounts for the largest amount of variance in the data. Factors that explain the least amount of variance are discarded. In this study, factors with eigenvalues greater than 1 were retained [27].

To allow for better differentiation of the factors, factor rotation was utilised. Orthogonal rotation results in independent factors; oblique rotation allows the factors to correlate. As there was no consensus as to whether or not the underlying factors should be related, both direct oblimin (oblique) and varimax (orthogonal) rotation were employed.

For interpretation purposes, the weights (loadings) of the items for each factor are considered, i.e. items having a large weight are used to label a factor. In a factor analysis, these loadings describe the strength of the relationship between each MDADI question and the underlying factor. Factor loadings were initially interpreted with an absolute value greater than 0.5 [28]. Recognising that a factor loading threshold greater than 0.5 is acceptable whilst one that is greater than 0.7 is deemed good [29], a more stringent factor loading threshold of greater than 0.7 was used to identify the top endorsed MDADI items.

The Kaiser–Meyer–Olkin measure of sampling adequacy and Bartlett’s test were generated to ensure that the criteria for a satisfactory factor analysis were met [24].

As highlighted by a recent systematic review by Patel et al. [30], the MDADI did not include a plan for missing data. Missingness in this study was assessed by evaluation of the percentage of questionnaire responses missing for each item. Participant anonymisation at previous enrolment to the databases precluded a statistical assessment of differences in demographic and clinical characteristics between those who submitted complete and incomplete MDADI questionnaires.

The top five endorsed items using PAF and PCA were checked for agreement and descriptive statistics for each item were generated. Cronbach’s alpha was used to evaluate the psychometric properties, reliability, and internal consistency of the MDADI, with an acceptable value ranging from 0.7 and 0.9, where higher values would suggest redundancy of items [31]. Preliminary validity assessment was also carried out with a view to future expansion.

## Results

### Patient Characteristics

There was a total of 239 patients identified from the three databases. Of the 216 patients with complete treatment data, there were 30 patients in database one (the minimally invasive surgical (TLM/TORS) group), 142 patients in database two (the non-surgical (CRT/RT) group), and 44 patients in database three (the feeding tube group (NGT/RIG)). Patient demographics for the entire cohort are summarised in Table 1.

**Table 1:** Patient demographics

Parameter	Patients (*n*)	Percent (%)
Total	239	100
Gender		
Male	191	79.9
Female	48	20.1
Site		
Oropharyngeal	93	38.9
Hypopharyngeal	32	13.4
Laryngeal	82	34.3
Nasopharyngeal	10	4.2
Unspecified	22	9.2
T stage		
T1	49	21.1
T2	46	19.8
T3	52	22.4
T4	63	27.2
Tx	22	9.5
N stage		
N0	88	40
N1	52	23.6
N2	73	33.2
N3	7	3.2
Age		
Minimum	41	
Maximum	89	
Mean	63.3	

### MDADI Responses

Of the total MDADI responses, 20 questionnaires had missing values. No more than 5% of questionnaire responses were missing per item (Table 2). After removing the 20 missing questionnaires, 196 questionnaires were available for the factor analysis. The 3-month composite MDADI scores ranged from 22.1 to 100, and the mean composite score was 68.6. The internal consistency of the MDADI questionnaire responses was assessed using the Cronbach’s alpha statistic. Cronbach’s alpha was 0.939 for all questions combined, excluding the global item question: “My swallowing ability limits my day-to-day activities”.

**Table 2:** Missing values analysis

Univariate statistics							
	N	Mean	Std Deviation	Missing		No. of extremes^a^	
				Count	Percent	Low	High
Activity	215	3.15	1.342	1	.5	0	0
Embarrassed	214	3.60	1.145	2	.9	14	0
Cooking	214	3.43	1.315	2	.9	0	0
End of Day	212	3.42	1.180	4	1.9	0	0
Conscious	213	3.08	1.241	3	1.4	0	0
Upset	215	3.12	1.351	1	.5	0	0
Effort	215	3.04	1.356	1	.5	0	0
Go Out	216	3.83	1.118	0	.0	32	0
Income	212	3.86	.135	4	1.9	27	0
Longer	211	2.52	1.292	5	2.3	0	0
Why	213	3.44	1.154	3	1.4	0	0
Irritate	214	3.81	.952	2	.9		
Cough	213	3.30	1.278	3	1.4	0	0
Social	213	3.35	1.307	3	1.4	0	0
Eat Out	214	3.07	1.311	2	.9	0	
Limit	214	3.05	1.328	2	.9	0	0
Weight	214	3.35	1.192	2	.9	0	0
Esteem	214	3.60	1.158	2	.9	19	0
Amount	210	3.53	1.107	6	2.8	10	0
Excluded	213	3.67	1.156	3	1.4	17	0

^a^Number of cases outside the range (Q1–1.5*IQR, Q3 + 1.5*IQR)

Q1 = first quartile, Q3 = third quartile, IQR = interquartile range

### Initial Selection of Factors

**Fig. 1:**
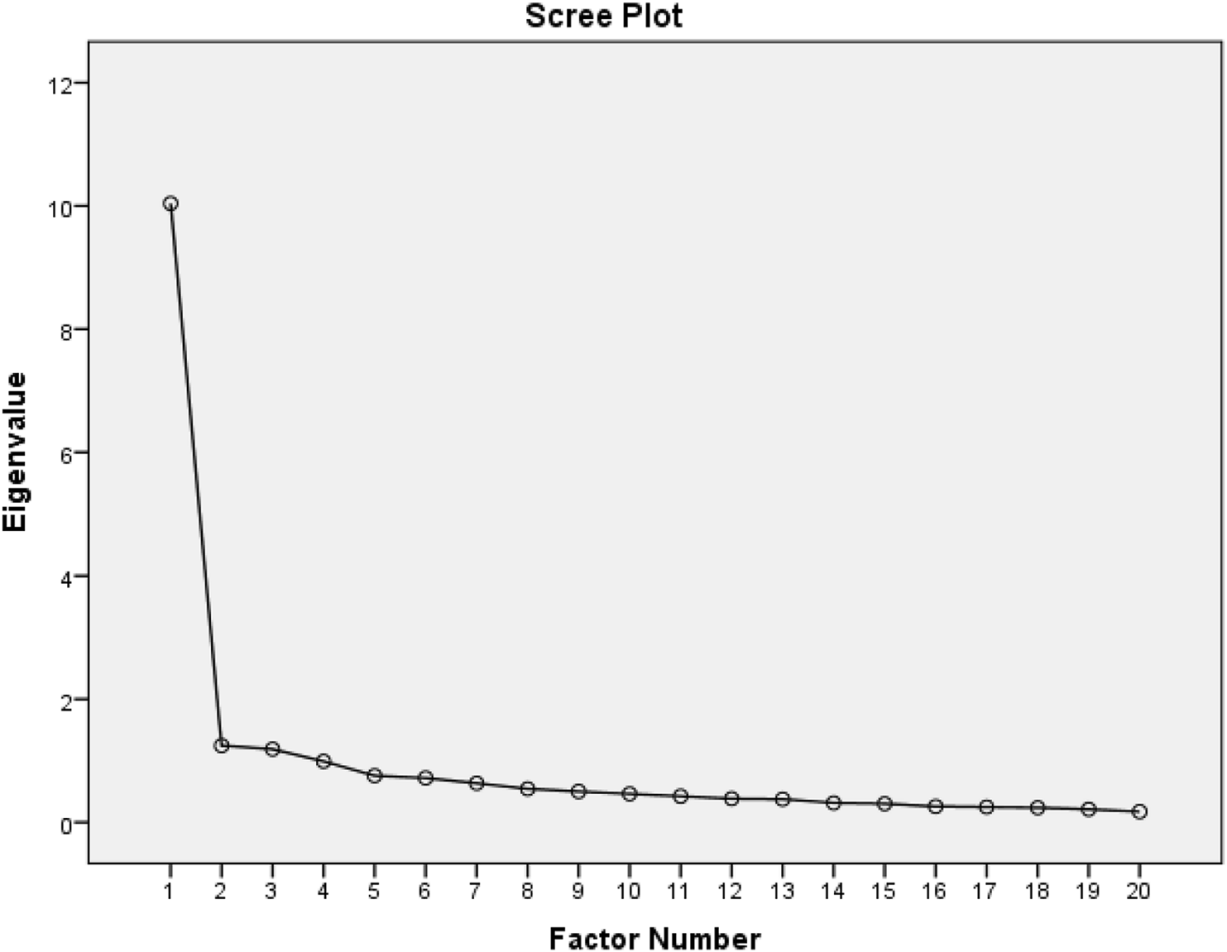
Scree plot of MDADI factors. MDADI factors shown along X-axis and corresponding eigenvalues along y-axis. Factors 1–3 had eigenvalues greater than 1

Factors were initially selected having eigenvalues greater than 1. Three factors met this criterion (Fig. 1). The percentage of variance in item responses accounted for by the first, most important, factor was around 50% (before rotation). Items having large loadings within these three factors (after rotation) were further examined.

### Principal Axis Factoring (PAF)

**Fig. 2:**
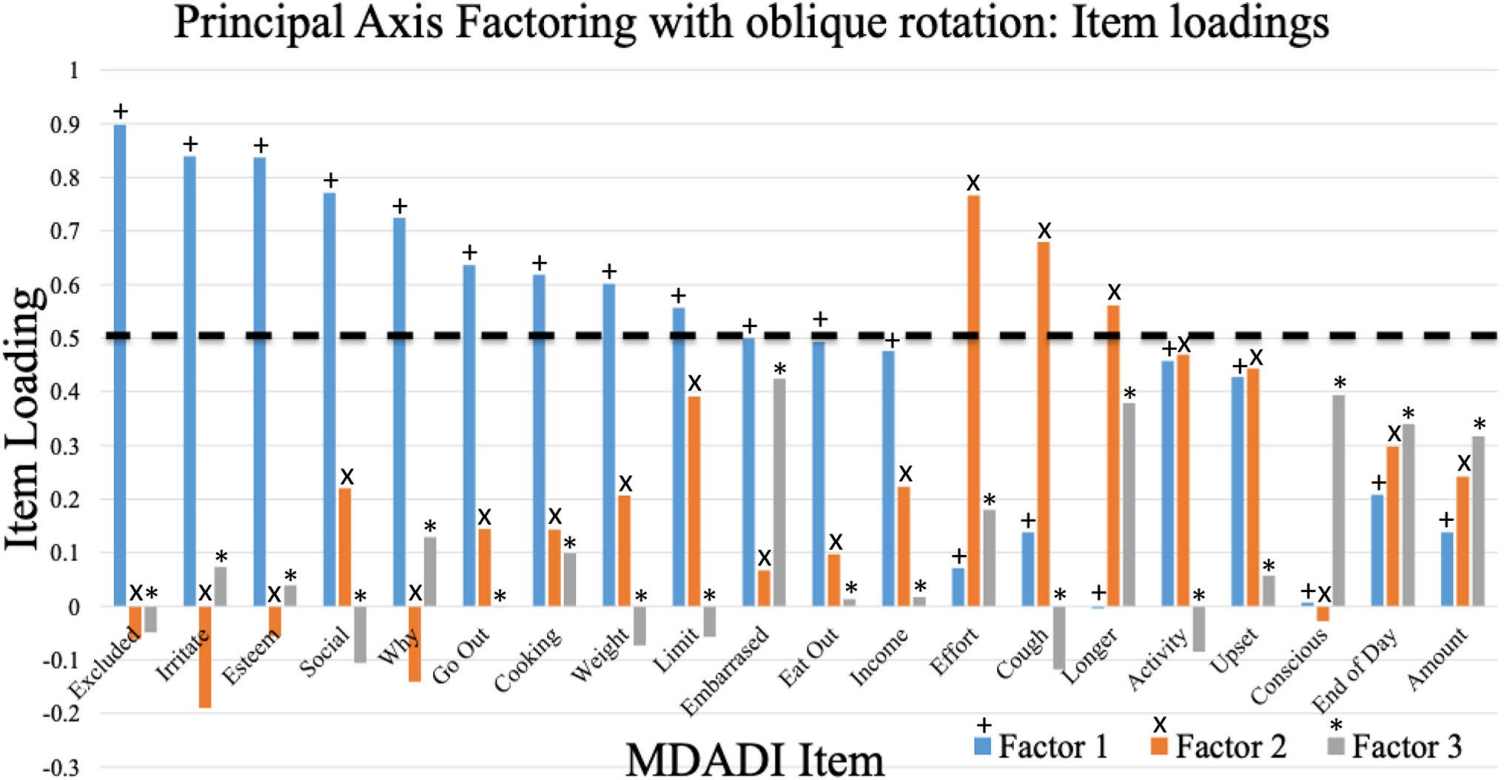
Examination of factor loadings greater than a 0.5 threshold

**Fig. 3:**
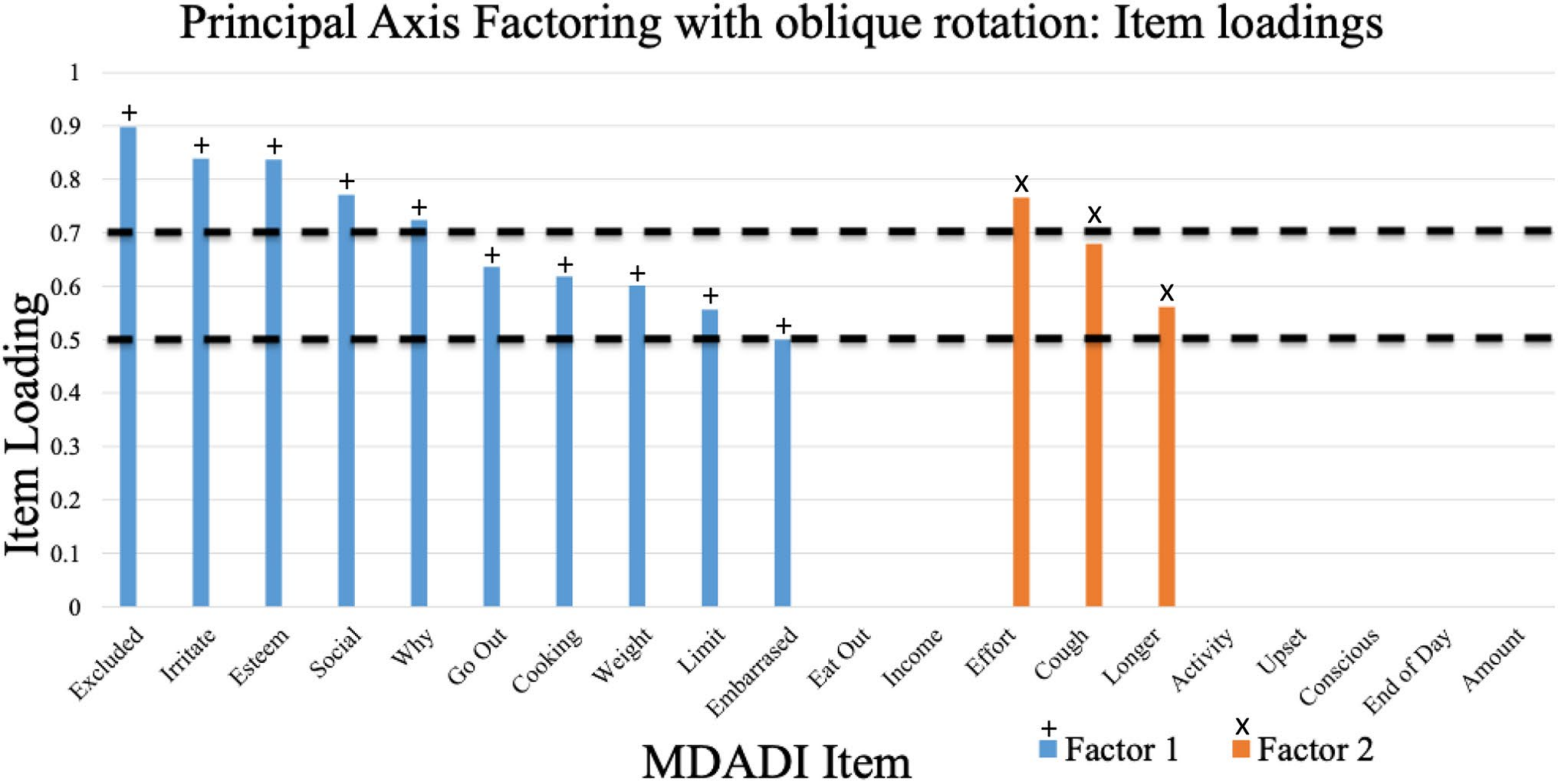
Identification of a factor with stringent item loadings greater than a 0.7 threshold

Principal axis factoring (PAF) was performed with oblique rotation and the loading for each item was observed within the three retained factors (Fig. 2). We then identified loadings over the 0.5 threshold (in absolute value). The top loading items occurred within the first two factors (Fig. 3).

### Top Endorsed MDADI Items

When a more stringent loading threshold of 0.7 is taken, the five top MDADI items in the first, most important, factor were identified, and these are summarised in Table 3. When compared to their original subscales, two of the items are functional, two are emotional, and one is related to a physical subscale.

**Table 3:** Top 5 MDADI items from factor 1 ranked in order of loading and matched to the original MDADI subscales

Factor 1 Loading (Rank)	MDADI Question	Subscale
0.898 (1)	I feel excluded because of my eating habits	Functional
0.839 (2)	Other people are irritated by my eating problem	Emotional
0.837 (3)	I have low self-esteem because of my swallowing problem	Emotional
0.771 (4)	My swallowing problems limit my social and personal life	Functional
0.724 (5)	People ask me, "why can't you eat that?"	Physical

### Principal Components Analysis (PCA)

Factor analysis with principal components extraction was then performed with oblique and orthogonal rotation and compared to PAF with oblique and orthogonal rotation (Table 4). Based on factor loadings and a threshold of 0.7, the top five items in factor 1 for PCA were consistent with those of PAF, regardless of method of rotation.

**Table 4:** Comparison of MDADI factor 1 loadings for PAF and PCA

Extraction Method		PAF		PCA	
Rotation Method		Oblique	Orthogonal	Oblique	Orthogonal
MDADI Item	Abbreviation	Factor 1 Loadings			
I feel excluded because of my eating habits	Excluded F4	0.898	0.768	0.864	0.796
Other people are irritated by my eating problem	Irritate E3	0.839	0.709	0.912	0.794
I have low self-esteem because of my swallowing problem	Esteem E6	0.837	0.731	0.836	0.775
My swallowing problems limit my social and personal life	Social F3	0.771	0.710	0.705	0.722
People ask me, "why can't you eat that?"	Why P3	0.724	0.627	0.845	0.739

### Internal Consistency and Tests of Validity

The questionnaire responses from the top 5 items from factor 1 were assessed and found to have a Cronbach’s alpha of 0.9. Additionally, there was a significant difference when comparing the MDADI responses for the surgical (TLM/TORS) versus non-surgical (CRT/RT) groups averaged across the top five items in factor 1 (*p* = 0.0004, unpaired t-test). This differentiation between known groups is an indicator of construct validity [22]. Furthermore, association was established between (i) the mean score of the aforementioned two items from the functional subscale, (ii) the mean score of the aforementioned two items from the emotional subscale, and (iii) the score from the one item from the physical subscale and the functional, emotional, and physical subscale scores, respectively, of the original MDADI (Pearson’s correlation coefficient for factor 1 subscale mean scores versus corresponding original MDADI subscale scores: emotional, r = 0.85; functional, r = 0.93; and physical, r = 0.64), thus, providing evidence that a reduced instrument would be valid.

## Discussion

This study aimed to identify the most important underlying MDADI questionnaire construct using retrospective data from HNC patients and explored the potential to abbreviate the MDADI for clinical and research purposes. Our study evaluated 196 MDADI questionnaires from surgical and non-surgical HNC patients. We attempted to cover all HNC sites, stages, and treatment modalities, by including consecutive HNC patients with a spectrum of disease and treatment-related dysphagia severity.

By performing exploratory factor analysis on this cohort of MDADI responses, we found a single underlying questionnaire construct (factor) which remained consistent across all methods of statistical enquiry using principal components analysis and principal axis factoring. Throughout our statistical interrogation, the same 5 questions (loading onto the single factor) were repeatedly identified. The 5 questions (and their corresponding subscales) were

1. I feel excluded because of my eating habits (Functional)
2. Other people are irritated by my eating problem (Emotional)
3. I have low self-esteem because of my swallowing problem (Emotional)
4. My swallowing problems limit my social and personal life (Functional)
5. People ask me, “Why can’t you eat that?” (Physical)

These 5 questions appeared in the 3 different subscales of the original MDADI (emotional, functional, and physical), suggesting that they may all be related to the same underlying questionnaire construct.

Our results reveal that at three months post-treatment, when patients show the most deterioration of swallowing function, the most highly endorsed items are related to functional and emotional aspects rather than the physical aspects, in spite of the physical subscale having the most items, eight, compared with five and six for the functional and emotional subscales, respectively. This substantiates the MDADI questionnaire as a dysphagia-specific QOL scale rather than a patient reported symptom scale. Moreover, even the retained item in the physical subscale is related to a social context more than a physical impairment of swallowing (P3: People ask me: “Why can’t you eat that?”). The psychosocial impact of dysphagia is well documented in the literature, characterised by avoidance of social eating, anxiety over mealtimes, isolation and low self-esteem [32–35]. Items covered in this reduced MDADI represent these commonly reported problems, and we suggest referring to this abbreviated version as the “MiniDADI”.

In the original MDADI paper [8], the Cronbach alpha for the total MDADI score was 0.96, suggesting some redundancy of items within the questionnaire. The internal consistency for the top five endorsed items was 0.9 after factor analysis was applied in our study and was, therefore, deemed to be within the acceptable value range for the Cronbach’s alpha statistic. Thus, shortening the MDADI to 5 questions from 20 questions did not jeopardise its reliability in terms of internal consistency. Interestingly, the top 5 questions identified did not include the global question: “My swallowing ability limits my day-to-day activities” (abbreviated as “Limit”, Appendix A). A plausible explanation for this is that a large proportion of the variance in questionnaire responses could be accounted for by the top 5 questions in factor 1. The global question, although widely used in current practice as a quick measure of dysphagia, did not account for enough of the response variance to be used as a standalone discerning item from the MDADI questionnaires which were tested in this study.

Construction of the MiniDADI scores would follow the same procedure as that of the standard MDADI, i.e. multiplying the mean of the responses from the 5 included questions by 20, generating a score ranging from 20 representing a low QOL function to 100 indicating high QOL function.

## Limitations

This study has several limitations that should be noted. This retrospective analysis was limited in geographical generalisability as it only included patients who were treated at two centres in the northeast of England. The assessment of missingness, and hence potential bias in analysis, could only be performed by confirming that there was less than 5% of questionnaire responses missing per item. Because participant responses had already been anonymised at the time of enrolment to the databases, it was not possible to retrospectively analyse for any difference in the demographic and clinical characteristics between complete and incomplete questionnaire responders. Thus, it was only possible to partially confirm that incompleteness occurred at random due to the small proportion (< 5%) responses missing per item [36]. It should also be borne in mind that the analysis performed in this study was an exploratory factor analysis which identified and statistically justified a single underlying questionnaire construct (factor 1).

The MDADI questions abbreviated by “Effort” and “Cough” appear to have large loadings, but as seen on Figs. 2 and 3, and these items are predominant on factor 2. Thus, they represent a different (and less statistically important) “dimension” in the data. As already demonstrated, factor 1 is primarily associated with the social aspects of dysphagia whereas “Effort” and “Cough” are concerned with the physical and have not been included in the MiniDADI. In the future, however, the MiniDADI may be expanded and explored using two subscales – the first containing the aforementioned 5 items and the second having 2 items from the original MDADI physical domain.

It is recognised that Cronbach’s alpha is the only measure of reliability that has been conducted on the MiniDADI so far. Test–retest reliability will be carried out in the future. Differentiation by known groups is an indicator of construct validity [22], and in this study, it was indeed established that the mean 3-month MiniDADI scores for TLM/TORS (83.3) was significantly higher than CRT/RT (70.3) (*p* = 0.0004). Assessment of concurrent criterion validity and construct validity (both convergent and divergent) involves the correlation of MiniDADI scores with other measurements which are taken simultaneously. The MiniDADI is composed of items from the functional (2 items), emotional (2 items), and physical (1 item) MDADI subscales; upon investigation of the correlation between the associated three MiniDADI *subscale* scores and the corresponding three subscales of the original MDADI, strong correlation was found (Pearson’s correlation coefficient for MiniDADI subscale mean scores versus corresponding original MDADI subscale scores: emotional, r = 0.85; functional, r = 0.93; and physical, r = 0.64), thus, providing evidence that an abbreviated instrument is valid. In the future, evaluation of concurrent validity will be carried out using a separate cohort of HNC patients by looking at associations between the MiniDADI and a gold standard (e.g. Eating Assessment Tool (EAT-10) or Swallowing Quality of Life questionnaire (SWAL-QOL)); relationships between the MiniDADI and a related construct (e.g. Sydney Swallow Questionnaire) will be examined to assess construct validity. Further testing would also ensure that the MiniDADI score is sufficiently robust to be used in the assessment of minimal clinically important difference (MCID) [37]. The authors’ advise against clinical use of the MiniDADI until further validation is completed.

## Conclusion

Factor analysis performed on this group of patients has generated a model for the MDADI questionnaire which appears to be explained by a single underlying factor. The single factor isolated incorporates an overlap of 3 of the original MDADI subscales and implies some redundancy of questions in the MDADI. In the increasingly busy clinical HNC setting, it appears that the single underlying construct of the MDADI can be tested in a quick and robust manner by using the 5 top loading question items identified in factor 1.

PROs are an essential component of treatment and disease-related outcome assessment and should be collected at regular intervals, before and after treatment. Reliable, valid, and acceptable tools are required to reduce patient burden, increase response rate, and produce robust data to encourage more longitudinal assessments of dysphagia in HNC. This initial study identified that an abbreviated MiniDADI may be a suitable PRO, with further testing required to substantiate these findings before implementation in routine clinical practice.

## Appendix A

The M. D. Anderson Dysphagia Inventory (MDADI) with corresponding abbreviations and subscale.

**Table:**

Subscale MDADI question	(Abbreviation)
(G) My swallowing ability limits my day-to-day activities.	(Limit).
(E) I am embarrassed by my eating habits.	(Embarrassed).
(F) People have difficulty cooking for me.	(Cooking).
(P) Swallowing is more difficult at the end of the day.	(End of Day).
(E) I do not feel self-conscious when I eat.	(Conscious).
(E) I am upset by my swallowing problem.	(Upset).
(P) Swallowing takes great effort.	(Effort).
(E) I do not go out because of my swallowing problem.	(Go Out).
(F) My swallowing difficulty has caused me to lose income.	(Income).
(P) It takes me longer to eat because of my swallowing problem.	(Longer).
(P) People ask me, “Why can’t you eat that?”	(Why).
(E) Other people are irritated by my eating problem.	(Irritate).
(P) I cough when I try to drink liquids.	(Cough).
(F) My swallowing problems limit my social and personal life.	(Social).
(F) I feel free to go out to eat with my friends, neighbors, and relatives.	(Eat Out).
(P) I limit my food intake because of my swallowing difficulty.	(Limit).
(P) I cannot maintain my weight because of my swallowing problem.	(Weight).
(E) I have low self-esteem because of my swallowing problem.	(Esteem).
(P) I feel that I am swallowing a huge amount of food.	(Amount).
(F) I feel excluded because of my eating habits.	(Excluded).

## References

[1] Raber-Durlacher JE, Brennan MT, Verdonck-de Leeuw IM, Gibson RJ, Eilers JG, Dysphagia Section, Oral Care Study Group MA of SC in C (MASCC)/International S of OO (ISOO) (2012). Swallowing dysfunction in cancer patients. Support Care Cancer.

[2] Shune SE, Karnell LH, Karnell MP, Van Daele DJ, Funk GF (2012). Association between severity of dysphagia and survival in patients with head and neck cancer. Head Neck.

[3] Wilson JA, Carding PN, Patterson JM (2011). Dysphagia after nonsurgical head and neck cancer treatment: Patients’ perspectives. Otolaryngol - Head Neck Surg.

[4] Pedersen A, Wilson J, McColl E, Carding P, Patterson J (2016). Swallowing outcome measures in head and neck cancer - How do they compare?. Oral Oncol.

[5] Patterson JM, McColl E, Carding PN, Wilson JA (2018). Swallowing beyond six years post (chemo)radiotherapy for head and neck cancer; a cohort study. Oral Oncol.

[6] Martin A, Murray L, Sethugavalar B, Buchan C, Williams GF, Sen M (2018). Changes in Patient-reported Swallow Function in the Long Term After Chemoradiotherapy for Oropharyngeal Carcinoma. Clin Oncol.

[7] Rogers SN (2016). Improving quality-of-life questionnaires in head and neck cancer. Expert Rev Qual Life Cancer Care.

[8] Chen AY, Frankowski R, Bishop-Leone J, Hebert T, Leyk S, Lewin J (2001). The development and validation of a dysphagia-specific quality-of-life questionnaire for patients with head and neck cancer: the M. D. Anderson dysphagia inventory. Arch Otolaryngol Head Neck Surg.

[9] Dawe N, Patterson J, O’Hara J (2016). Functional swallowing outcomes following treatment for oropharyngeal carcinoma: a systematic review of the evidence comparing trans-oral surgery versus non-surgical management. Clin Otolaryngol.

[10] Roe JWG, Carding PN, Dwivedi RC, Kazi RA, Rhys-Evans PH, Harrington KJ (2010). Swallowing outcomes following Intensity Modulated Radiation Therapy (IMRT) for head & neck cancer - A systematic review. Oral Oncol.

[11] Owadally W, Hurt C, Timmins H, Parsons E, Townsend S, Patterson J (2015). PATHOS: A phase II/III trial of risk-stratified, reduced intensity adjuvant treatment in patients undergoing transoral surgery for Human papillomavirus (HPV) positive oropharyngeal cancer. BMC Cancer.

[12] Petkar I, Rooney K, Roe JWG, Patterson JM, Bernstein D, Tyler JM (2016). DARS: A phase III randomised multicentre study of dysphagia- optimised intensitymodulated radiotherapy (Do-IMRT) versus standard intensity- modulated radiotherapy (S-IMRT) in head and neck cancer. BMC Cancer.

[13] Nichols AC, Theurer J, Prisman E, Read N, Berthelet E, Tran E (2019). Radiotherapy versus transoral robotic surgery and neck dissection for oropharyngeal squamous cell carcinoma (ORATOR): an open-label, phase 2, randomised trial. Lancet Oncol.

[14] Hutcheson KA, Barrow MP, Barringer DA, Knott JK, Lin HY, Weber RS (2017). Dynamic Imaging Grade of Swallowing Toxicity (DIGEST): Scale development and validation. Cancer.

[15] Kulbersh BD, Rosenthal EL, McGrew BM, Duncan RD, McColloch NL, Carroll WR (2006). Pretreatment, preoperative swallowing exercises may improve dysphagia quality of life. Laryngoscope.

[16] Lu W, Wayne PM, Davis RB, Buring JE, Li H, Goguen LA (2012). Acupuncture for dysphagia after chemoradiation in head and neck cancer: Rationale and design of a randomized, sham-controlled trial. Contemp Clin Trials.

[17] Patterson JM, Exley C, McColl E, Breckons M, Deary V (2018). Feasibility and acceptability of combining cognitive behavioural therapy techniques with swallowing therapy in head and neck cancer dysphagia. BMC Cancer.

[18] Ryu JS, Kang JY, Park JY, Nam SY, Choi SH, Roh JL (2009). The effect of electrical stimulation therapy on dysphagia following treatment for head and neck cancer. Oral Oncol.

[19] Goepfert RP, Lewin JS, Barrow MP, Gunn GB, Fuller CD, Beadle BM (2017). Long-Term, Prospective Performance of the MD Anderson Dysphagia Inventory in “Low-Intermediate Risk” Oropharyngeal Carcinoma After Intensity Modulated Radiation Therapy. Int J Radiat Oncol.

[20] Roe JWG, Drinnan MJ, Carding PN, Harrington KJ, Nutting CM (2014). Patient-reported outcomes following parotid-sparing intensity-modulated radiotherapy for head and neck cancer. How important is dysphagia?. Oral Oncol.

[21] Rolstad S, Adler J, Rydén A (2011). Response burden and questionnaire length: Is shorter better?. A review and meta-analysis Value Heal.

[22] Boateng GO, Neilands TB, Frongillo EA, Melgar-Quiñonez HR, Young SL (2018). Best Practices for Developing and Validating Scales for Health, Social, and Behavioral Research: A Primer. Front Public Heal.

[23] Chatfield C, Collins AJ (1991). Introduction to Multivariate Analysis. Routledge.

[24] Field A (2013). Discovering statistics using IBM SPSS Statistics: and sex and drugs and rock ‘n’ roll.

[25] Nunnally JC (1978). Psychometric Theory.

[26] Stevens J (2020). Applied Multivariate Statistics for the Social Sciences.

[27] Kaiser HF (1960). The application of electronic computers to factor analysis. Educ Psychol Measur.

[28] Manly BF (1997). Multivariate Statistical Methods: A Primer.

[29] Hutcheson G (1999). The Multivariate Social Scientist.

[30] Patel DA, Sharda R, Hovis KL, Nichols EE, Sathe N, Penson DF (2017). Patient-reported outcome measures in dysphagia: A systematic review of instrument development and validation. Dis Esophagus.

[31] de Vet HCW, Terwee CB, Mokkink LB, Knol DL (2011). Measurement in Medicine.

[32] García-Peris P, Parón L, Velasco C, de la Cuerda C, Camblor M, Bretón I (2007). Long-term prevalence of oropharyngeal dysphagia in head and neck cancer patients: Impact on quality of life. Clin Nutr.

[33] Thankappan K, Iyer S, Menon JR (2018). Dysphagia Management in Head and Neck Cancers.

[34] Nguyen NP, Frank C, Moltz CC, Vos P, Smith HJ, Karlsson U (2005). Impact of dysphagia on quality of life after treatment of head-and-neck cancer. Int J Radiat Oncol Biol Phys.

[35] Silveira M, Dedivitis R, Queija D, Nascimento P (2014). Quality of Life in Swallowing Disorders after Nonsurgical Treatment for Head and Neck Cancer. Int Arch Otorhinolaryngol.

[36] Bland M. Martin Bland (2015): An introduction to medical statistics. 4th ed. Oxford University Press; 2015.

[37] Hutcheson KA, Barrow MP, Lisec A, Barringer DA, Gries K, Lewin JS (2016). What is a clinically relevant difference in MDADI scores between groups of head and neck cancer patients?. Laryngoscope.

